# The COVID-19 Vaccination and Vaccine Inequity Worldwide: An Empirical Study Based on Global Data

**DOI:** 10.3390/ijerph19095267

**Published:** 2022-04-26

**Authors:** Chuanlin Ning, Han Wang, Jing Wu, Qinwei Chen, Huacheng Pei, Hao Gao

**Affiliations:** 1 School of Media and Communication, Shanghai Jiao Tong University, Shanghai 200240, China; ningchuanlin@sjtu.edu.cn; 2 School of Journalism and Communication, Jinan University, Guangzhou 510632, China; jacobwang0606@163.com; 3 Faculty of Social Sciences, University of Ljubljana, 1000 Ljubljana, Slovenia; jw0822@student.uni-lj.si; 4 School of Journalism and Communication, Nanjing Normal University, Nanjing 210097, China; cqinwei2022@163.com; 5 School of Economics and Finance, Shanghai International Studies University, Shanghai 200083, China; 0203700657@shisu.edu.cn

**Keywords:** the COVID-19 vaccination, vaccine inequity, immunization coverage, public health communication

## Abstract

Vaccination is critical for controlling the COVID-19 pandemic. However, the progress of COVID-19 vaccination varies from different countries, and global vaccine inequity has been a worldwide public health issue. This study collected data from the Our World in Data COVID-19 vaccination data set between 13 December 2020 and 1 January 2022. The measurement reflecting the pandemic situation included New cases, New deaths, Hospital patients, ICU patients, and the Reproduction rate. Indicators for measuring the vaccination coverage included Total vaccinations per hundred and People vaccinated per hundred. The Human Development Index (HDI) measured the country’s development level. Findings indicated that countries with higher HDI have more adequate vaccine resources, and global vaccine inequity exists. The study also found that vaccination significantly mitigates the pandemic, and reaching 70% immunization coverage can further control the epidemic. In addition, the emergence of Omicron variants makes the COVID-19 epidemic situation even worse, suggesting the importance and necessity of addressing vaccine inequity. The globe will face a greater challenge in controlling the pandemic if lower-vaccinated countries do not increase their vaccination coverage. Addressing the issue of vaccine inequity needs the cooperation of HIC, LMIC, public health departments, and vaccine producers. Moreover, the media has to contribute to effective public health communication by raising public perceptions of the COVID-19 pandemic, vaccination, and vaccine inequity.

## 1. Introduction

Vaccination is an essential intervention for controlling infectious diseases, reducing the disease burden among vaccinated individuals, and reducing the spread of infection to unvaccinated groups [1]. Vaccine equity means that vaccines should be allocated across all countries based on needs and regardless of their economic status [2]. According to WHO, the vast majority of COVID-19 vaccines have been administrated in high-and upper-middle-income countries [3]. However, people in low-income countries did not share equal access to the vaccines, which indicates the potential vaccine inequity. The vaccine equity program advocated by the WHO emphasized the importance of vaccination coverage in mitigating the COVID-19 epidemic [3]. Thus, the vaccine inequity in this study refers to the inequity of vaccination coverage. However, vaccine inequity has persisted since the World Health Assembly (WHA) began its global roll-out of immunization programs in 1974 [4]. A wide disparity of routine immunization coverage has emerged between high- and low-income countries in the 1980s [5]. Reports showed that low–middle-income countries (LMIC) bear a higher burden of vaccine-preventable diseases [6]. Global vaccine inequity has been a worldwide public health issue.

Mass COVID-19 vaccination has been considered an economical and effective measure to mitigate the epidemic since the worldwide outbreak of COVID-19 in December 2019 [7]. Countries worldwide began implementing COVID-19 vaccination program by the end of 2020. However, the progress of promoting COVID-19 vaccines across the world is inconsistent due to differences in national economy and vaccine development technology among countries, also with significant differences in vaccination coverage. By 2020, Canada had purchased enough doses to vaccinate their entire population more than four times, and the UK had bought doses for the people to vaccinate three times. As of 4th March 2021, more than 80 countries had not yet administered one-shot [8]. A study on the vaccination data set of 178 countries until 31 March 2021 found that 80% of the world’s population in developing countries had only about 5% of the world’s COVID-19 vaccines, while the remaining 20% of the world’s population in developed countries had about 95% of the world’s COVID-19 vaccines [9], indicating a severe global COVID-19 vaccine inequity.

In response to a more equitable distribution of vaccination globally, the World Health Organization (WHO) has called to solve the COVID-19 vaccine inequity worldwide and improve vaccination difficulties in LMIC [10]. In April 2020, CEPI, Gavi, and WHO jointly launched the COVAX program to address the COVID-19 vaccine inequity [11], and UNICEF joined as a partner in February 2021 [12]. The COVAX program aims to promote the development and production of the COVID-19 vaccine and ensure fair and equitable access to the vaccine in every country [10]. With the facilitation of WHO and the collaboration of high-income countries (HIC) and vaccine manufacturers, the program has administrated nearly one billion doses of vaccines to 144 countries [13]. However, low-income regions, accounting for 8% of the worlds’ population, have been allocated less than 1% of the total global vaccine doses [14].

Public health experts estimated that global herd immunity with approximately 70% of the 7.9 billion people worldwide vaccinated could control the COVID-19 pandemic [15]. COVID-19 vaccine inequity brings more severe pressure to low-vaccination countries and further negatively influences high-income countries. A study based on data modeling revealed that differences in the distribution of the COVID-19 vaccines between LMIC and HIC would lead to shorter intervals and increase the infected cases in each wave of the pandemic outbreak [16]. According to Tedros Adhanom Ghebreyesus [9], WHO Director-General, vaccine inequity allows the continued spread of the COVID-19 and creates space for mutations in the virus, which will reduce the effectiveness of the COVID-19 vaccines. Given the uneven vaccination rate across the globe and the emergence of more infectious COVID-19 variants, achieving “herd immunity” is becoming increasingly difficult [17]. In other words, all countries around the world would not escape from the COVID-19 threat as long as the epidemic remains in any corner of the world [18].

The world is still facing the challenges brought by the pandemic, and people’s lives are being affected by it. Since the COVID-19 vaccination started at the end of 2020, what is the current vaccination progress in the globe? What are the differences in the COVID-19 vaccination among countries at different development levels? This study collected global data about the COVID-19 vaccination, the epidemic situation, and the national development levels as of 1 January 2022, exploring the following questions based on data analysis:

Q1: What is the overall situation of COVID-19 vaccination globally? Does the COVID-19 vaccine inequity still exist?

Q2: Has the global COVID-19 vaccination effectively mitigated or controlled the epidemic based on the data analysis?

## 2. Materials and Methods

### 2.1. Design and Data

This study collected data from the Our World in Data COVID-19 vaccination data set. This data set is a representative and official global data set on the epidemic situations and the COVID-19 vaccination progress, derived from national health departments, government reports, and official social media accounts. The data set continues to track the COVID-19 vaccination data for 225 countries since the first release of vaccination data on 13 December 2020, showing a snapshot of the scale and pace of vaccination progress in each country.

In terms of data processing, this study determined the time period of selected samples firstly. The earliest traceable data on the COVID-19 vaccination was on 13 December 2020, while the actual start of the COVID-19 vaccination in most countries was concentrated in 2021. This study targeted the sample from 13 December 2020 to 1 January 2022, investigating the vaccination situation during the period. Particularly, different models of data analysis used different data periods. Model 1 adopted the cross-sectional data of 1 January 2022. Results shown in Model 2, Model 3, and Tables 1 and 2 were calculated based on the data from 13 November 2020 to 1 January 2022, on the layer of day. Then, to compare differences in vaccination at the country level, this research retained data at the level of countries (regions) and excluded continent-level data. All data processing and analysis of this study were operated through Excel and STATA.

### 2.2. Regression Model

This study mainly explored the vaccination coverage and the COVID-19 epidemic situation in global.

#### 2.2.1. Vaccination

Although the WHO advocated equitable sharing of vaccine stocks at the very beginning, the actual COVID-19 vaccine stockpiles and vaccination progress varied widely between countries [19], and HIC always had more vaccines [20]. When studying the inequity of vaccine promotion in different cities in the US, DiRago et al. [8] used the proportion of the vaccinated population as a proxy for vaccine promotion. They found that vaccination increased less in communities with lower socioeconomic levels than in affluent communities.

Referring to the research method used by Basak et al. [21], this study collected the cross-sectional data on 1 January 2020 to explore the relationship between global vaccination and HDI, establishing the regression model (1):

(1) $$Vaccination_{i}=\alpha_{0\;}+\alpha_{1}HDI_{i}+\varepsilon_{i\;}\#$$

In Equation (1), Vaccination refers to the vaccination of Country *i* on 1 January 2022. Referring to the research conducted by Bhutto [20] and DiRago et al. [8], this study selected Total vaccinations per hundred and People vaccinated per hundred to represent the Vaccination. HDI is the Human Development Index.

#### 2.2.2. The COVID-19 Epidemic Situation

Ahmed [22] showed differences in detection, infection, hospitalization, and mortality across countries during the COVID-19 pandemic. This study developed the regression model (2) to test the COVID-19 epidemic situation:

(2) $$\mathrm{COVID}\text{-}19\;epidemic\;situation=\beta_{0}+\beta_{1\;}People\;vaccinated\;per\;hundred_{i,t}+\varepsilon_{i,t\;}\#$$

COVID-19 epidemic situation in Equation (2) indicates the COVID-19 epidemic situation in country = *i* at the time t. According to Ahmed’s [22] research, we selected New cases per million, New deaths per million, Hosp patients per million, and ICU patients per million as the indicators measuring the COVID-19 epidemic situation. As an independent variable, People vaccinated per hundred shows the number of people vaccinated in country *i* at the time *t*.

However, the virus has been evolving since the outbreak of COVID-19. More virulent and faster spreading mutant strains occurred, such as Omicron, discovered in South Africa on 9 November 2021. Omicron may evade current vaccine defenses and cause further spread of the epidemic [23,24,25]. Considering the COVID-19 epidemic situation might be different after Omicron occurred, this study conducted additional tests on Model (2) with the cut-off point of when Omicron was discovered. The additional model tests examined the relationship between the vaccination coverage and the COVID-19 epidemic situation before and after the emergency of Omicron.

Public health experts estimated that herd immunity will be achieved when 70% of the global population has been vaccinated [16]. Thus, this study established the dummy variable Seventy to test the immunization coverage, setting Seventy = 1 when People vaccinated per hundred was more than or equal to 70; otherwise, Seventy = 0. Here is the regression model (3):

(3) $$Immunization\;Coverage_{i,t}=\gamma_{0}+\gamma_{1\;}Seventy_{i,t}+\varepsilon_{i,t\;}\#$$

Immunization Coverage in Equation (3) represents the immunization coverage of country *i* at the time *t*. Similar to Model (2), the five specific indicators in Model (3) are New cases per million, New deaths per million, Hosp patients per million, ICU patients per million, and Reproduction rate.

### 2.3. Selection of Variables

Among the selected variables in the regression models, some are used as dependent and independent variables in different models. Moreover, all quantitative variables like the number of vaccines and people have been adjusted for the national population to ensure that the selected variables are comparable across countries. Furthermore, this study retained two orders of magnitude of adjustment (per hundred and per million) to make the correlation coefficients in regression results more intuitive.

#### 2.3.1. Interpretation of Indicators in Model (1)

Table 1 explains the definition of all variables in this study.

##### Establishment of Indicators

The numbers of national populations and vaccines vary in countries worldwide, so we adjusted the indicators at the population dimension to make the global data comparable.

From the perspective of the number of vaccines and the number of people vaccinated, this study selected two indicators for the Vaccination variable: Total vaccinations per hundred and People vaccinated per hundred. “Total vaccinations per hundred” measures the number of vaccines in different countries, and the calculation is the number of vaccines owned or distributed in a country divided by the hundred population of the country. The higher value of Total vaccinations per hundred indicates a higher number of vaccines per capita in the country, which means the vaccine resources in the country are more abundant. “People vaccinated per hundred” measures the number of people vaccinated in the country, calculated as the number of people vaccinated in a country divided by the hundred population of the country. In addition, the Our World data set counted all people with vaccination records in this indicator without distinguishing between the first, second, and third doses. Thus, this indicator does not distinguish between the number of vaccinated doses. The higher value of People vaccinated per hundred means greater vaccination coverage and adequate vaccine resources in a country. However, the two indicators did not appear continuously in the original data set because the data are not updated at the same time for each country. On this occasion, this study processed the two indicators manually. Starting with the first occurrence of vaccination data in a country, if there are no new data on the day t, we will use the last updated vaccination data before the date *t* to represent the vaccination situation of *t*.

This study selected HDI as the indicators of measuring the development levels of countries. HDI is a comprehensive indicator developed by the United Nations Development Program (UNDP) to assess human development in different countries, based on the critical points of health, education, and economic development [26]. The Human Development Index (HDI) is a summary measure of achievements in three key dimensions of human development: a long and healthy life, access to knowledge, and a decent standard of living. The HDI is the geometric mean of standardized indices for the dimension of health, education, and living standard [27]. Life expectancy (20–85 years) is the indicator for measuring the health dimension, and the data for this indicator are derived from UNDESA (2019). Expected years of schooling (0–18 years) and Mean years of schooling (0–15 years) are the indicators for measuring the education dimension. Data for Expected years of schooling are collected from UNESCO Institute for Statistics (2020), ICF Macro Demographic and Health Surveys (2008–2020), United Nations Children’s Fund (UNICEF) Multiple Indicator Cluster Surveys (2008–2020), and OECD (2019). Data for Mean years of schooling are from UNESCO Institute for Statistics (2020), Barro and Lee (2018), ICF Macro Demographic and Health Surveys (2008–2020), UNICEF Multiple Indicator Cluster Surveys (2008–2020), and OECD (2019). Living GNI per capita is an indicator of the living standard, and the data are from the World Bank (2020), IMF (2020), and United Nations Statistics Division (2020) [27].

The UNDP has assessed the human development levels of 189 countries in the world based on the HDI since 1990 and has classified these countries into four levels, namely, Very High Human Development (HDI ≥ 0.8), High Human Development (0.8 > HDI ≥ 0.7), Medium Human Development (0.7 > HD I≥ 0.55), and Low Human Development (HDI < 0.55) [28]. This indicator, which provides a more comprehensive picture of the human development level of a country than other single indicators, is widely used in scientific research to test correlations with other indicators [29].

This study described the COVID-19 epidemic situation from five aspects: new confirmed cases, new deaths, cases in hospital, cases in ICU, and the reproduction rate of the virus. The specific five indicators are named as New cases per million, New deaths per million, Hosp patients per million, ICU patients per million, and Reproduction rate. In addition to the Reproduction rate, the other four indicators share a similar calculation: the number of cases divided by the million population of a country. On the one hand, the numbers of new confirmed cases, new deaths, cases in hospital, and cases in ICU are of a great order of magnitude different to that of the total population of the country. Thus, we chose the million population as the denominator to make the indicator describing the COVID-19 epidemic situation more numerically intuitive. On the other hand, the results described with ‘per million’ show intuitive correlation coefficients of the regression analysis in the subsequent empirical tests. As a flow indicator, New cases reflects the increasing trend of confirmed cases and the dynamics of the epidemic. If the value of this indicator is smaller and gets closer to zero, the epidemic is better controlled. New deaths per million, Hosp patients per million, and ICU patients per million reflect the severity of the pandemic. New deaths per million measures the serious consequences of the epidemic, presenting the dynamic of death over time. A smaller value, even zero, indicates the good treatment of confirmed cases and the reduction of death risks. The number of ICU patients in ‘Our World in Data’ measures the impact of the epidemic on people, and the greater number of ICU patients represents the more serious pandemic. A smaller value of this indicator means that the deterioration of the epidemic are better controlled. Reproduction rate in ‘Our World in Data’ is an important indicator indicating the extent of the development of the epidemic. The reproduction rate represents the average number of new confirmed cases caused by a single infected individual. If the rate is greater than 1, the infection can spread in the population; if the rate is less than 1, the number of confirmed infections will decrease to zero [28]. Reproduction rate reflects the degree of epidemic control and the development trend of the pandemic to some extent. Hence, this study used this indicator to examine the epidemic’s situation and the development trend in different countries.

We selected People vaccinated per hundred to measure the vaccination rate. This indicator covers a wider range than the indicator of People fully vaccinated per hundred. In addition, it is reported that people will get a certain defense to the virus once vaccinated, no matter how many doses they have received. Moreover, the interval between each vaccination dose is long, which is difficult for statistics. In addition, we used a dummy variable, Seventy, to represent immunization coverage and test the achievement of herd immunity. In this study, we defined Seventy = 1 if the value of People vaccinated per hundred ≥70 and Seventy = 0 if the value of People vaccinated per hundred <70. The indicator Seventy is a supplementary test for the vaccination rate when reaching a certain coverage.

### 2.4. Methods

#### 2.4.1. OLS

This study used cross-sectional data and OLS regression in the model (1). Due to the limitation of HDI, some countries and regions like some island countries and Macau are not covered in the original data set; thus, the test sample of the model (1) includes 184 countries and regions.

#### 2.4.2. Fixed Effects

In Model (2) and Model (3), this study used country-data panel data and panel fixed effects for the regression analysis. The data sample in this part covers 225 countries and regions and is unbalanced panel data as each country started COVID-19 vaccination at different times. Regarding the large observation of the sample, this study took measures to avoid the potential temporal and spatial correlation. On the one hand, this study took 10 percent random samples of the total data sets for each regression by country classification. On the other hand, we used a Durbin–Watson test to avoid the autocorrelation of each variable. In addition, this study conducted additional analysis on random sub-samples of different percentile sizes to ensure the scientific of the results. This paper did not show the additional regression result of the random sub-samples due to the words limit, but we kept it for reference.

## 3. Results

### 3.1. Ordinary Least Squares Regression (OLS)

Table 2 shows the OLS test result of Model (1). Total vaccinations per hundred and People vaccinated per hundred which represent vaccination, positively correlated with HDI, significant at the 0.001 level. The finding indicates that vaccination varies by countries depending on the HDI level, and higher HDI means adequate vaccination.

**Figure 1:**
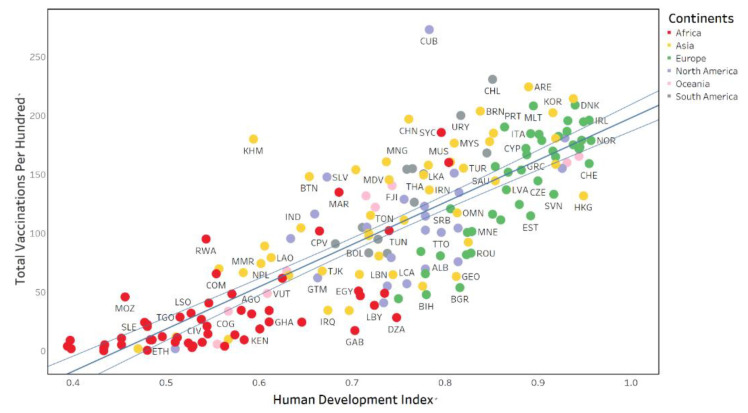
Total vaccinations per hundred and HDI in global countries.

**Figure 2:**
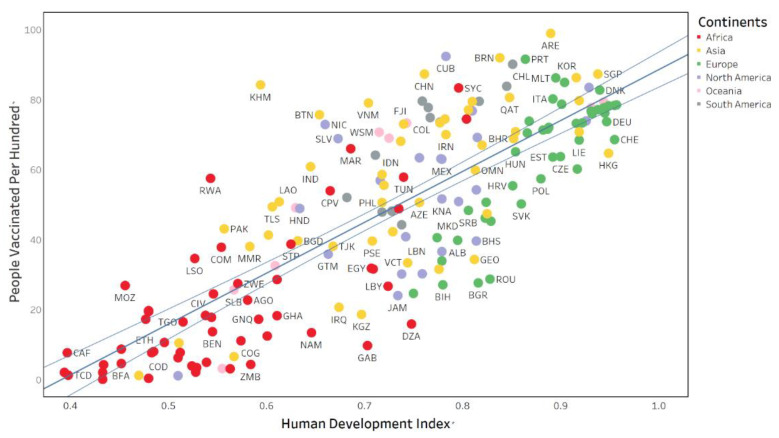
People vaccinated per hundred and HDI in global countries.

In addition, Figure 1 and Figure 2 below visually show the relationship between HDI and the two indicators of Vaccination. The two figures share some commonalities. First, the vaccination is positively correlated with HDI, and the number of vaccines per hundred and people vaccinated per hundred in a country increases as HDI increases. Second, the green dots representing European countries are mainly located in the upper right of the figures, indicating that European countries have higher HDI values and more adequate vaccine resources. Third, the red dots representing African countries are mainly on the bottom left of the figures, showing African countries with lower HDI values and fewer vaccine resources. Fourth, the middle of the figures is dominated by yellow dots (Asian countries) and gray dots (South American countries), which have moderate HDI levels and vaccination levels.

### 3.2. Fixed Effects Model Test

This study conducted a fixed-effects test on Model (2), and Table 3 shows the regression analysis results. The regression coefficients for New cases per million, Reproduction rate, were plus, while the regression coefficients for New deaths per million, Hosp patients per million, ICU patients per million were minus, being significant at the 0.001 level. The results showed that the increased vaccination rate significantly suppressed New deaths per million, Hosp patients per million, and ICU patients per million during the period of the sample, indicating the effectiveness of the COVID-19 vaccine in the three indicators. However, the results also showed that the COVID-19 vaccine could not suppress the New (confirmed) cases and Reproduction rate.

Then, this study conducted the fixed effects test of the model (3), and Table 4 shows the results. The relationship between the immunization coverage and other indicators is similar to Table 3. The regression coefficients of New cases per million and Reproduction rate were significantly positive at the 0.001 level, while the regression coefficients of New deaths per million and ICU patients per million were significantly negative at the 0.001 level. The results showed that a country reaching the herd immunity standard has a lower proportion of new dead cases, cases in hospital, and severe cases, but the immunization coverage (People vaccinated per hundred ≥70) cannot suppress the new confirmed cases and reproduction rate of the virus.

The test results of Model (3) and Model (4) showed that vaccination did not suppress the new confirmed cases and the reproduction rate of the virus. Studies have found that the effects of vaccination on suppressing the virus development would decline or even disappear over time, and the emergence of new mutants is one of the major reasons [30]. Thus, we performed an additional test on two groups, which were divided based on the date when Omicron appeared (9 November 2021).

Table 5 shows the regression results of the separate groups’ tests of Model (2), only listed with the regression coefficients of vaccination rate for simplification. Panel A showed that the regression coefficients of New cases per million, Reproduction rate were significantly negative at the 0.05 level, and the regression coefficients of New deaths per million, Hosp patients per million, and ICU patients per million were significantly negative at the 0.001 level. The results suggested that the effect of vaccination was significant before the emergence of Omicron. The increased vaccination coverage significantly reduced New cases per million, New deaths per million, Hosp patients per million, and ICU patients per million and effectively suppressed the Reproduction rate.

Panel B shows the regression coefficients after 9 November 2021. The regression coefficients of New cases per million were significantly positive at the 0.01 level, and that of the Reproduction rate was significantly positive at the 0.001 level. The regression coefficient of ICU patients per million is positive but not significant in statistics. On the one hand, the results reflected that the emergence of Omicron brought a faster spread of the virus and makes the COVID-19 epidemic situation even worse. On the other hand, the finding indicated that the current vaccines have a certain time lag and need further development according to the mutation of the virus.

## 4. Discussion

### 4.1. Current Vaccination Situation: Vaccine Inequity Persists

The COVID-19 vaccination data recorded the situation from 13 December 2020 to 1 January 2022, and the global roll-out of the COVID-19 vaccine has been underway for more than one year. However, the empirical research showed significant differences in vaccination progress across countries. The data showed the coverage of the COVID-19 vaccination in different countries as of 31st December 2021, such as United Arab Emirates (98.99%), Brunei (91.93%), and Singapore (87.26%). In addition, there are some countries with lower vaccination coverage, such as Niger (5.67%), Nigeria (4.88%), and Burundi (0.03%) [31]. The regression analysis of this study further indicated that countries with higher HDI and economic development have significantly higher vaccination coverage than countries with lower HDI. The COVID-19 is a common public health issue faced by humankind and the COVID-19 vaccination is the most effective way of responding to the epidemic. However, there is still a significant COVID-19 vaccine inequity among countries.

The strength of a country’s economic development determines the level of financial investment a country can make in vaccine development, production, and promotion. As a comprehensive indicator containing economic development, educational attainment, and health standards, HDI represents a country’s overall strength. HDI also reflects the ability of a country to deal with the COVID-19 pandemic, especially in vaccine development, production, and promotion. UNICEF argued that the average cost of the COVID-19 vaccine ranges from USD2 to USD37 per dose, and the distribution cost per person is about USD3.7 [32]. The production cost is a heavy financial burden for LMIC. Furthermore, the vaccination program and the promotion of the COVID-19 vaccine are related to a country’s health system and public health beliefs. Hence, in addition to the limitations of economic development, the education level, and national health level also affect COVID-19 vaccination and promotion.

### 4.2. The Importance of Reducing Vaccine Inequity: A Data-Level Validation

This study found that the new dead cases, cases in hospital, and ICU cases of the epidemic decreased significantly as the vaccination rate increased globally. The results indicated the positive influence of vaccination on mitigating the global pandemic. Although some skeptics and conspiracy theorists still questioned the effectiveness of the COVID-19 vaccine nowadays [33], this study showed the usefulness of the vaccine for epidemic control from empirical research. On this occasion, reducing vaccine inequity is of great significance for controlling the epidemic.

Due to the higher vaccination coverage, HIC with higher HDI have the ability to control the number of New deaths and ICU patients and allow for an early return to regular socioeconomic activity. In contrast, LMIC with lower HDI would still suffer from a prolonged crisis caused by the epidemic and the risk of virus mutation without achieving the immunization barrier. It would further affect the global response to the epidemic and widen the global economic and social development gap.

Another result in this study indicated that the proportion of new dead cases, cases in hospital and ICU cases reduced significantly when the COVID-19 vaccination rate reached 70%. This finding echoed the 70% immunization coverage target established before by public health experts. This finding also suggested that countries with vaccination coverage below 70% are at higher risk than those over 70%, further demonstrating the importance of addressing vaccine inequity. The data until 1 January 2022 showed that 79 countries had reached 70% national vaccination coverage and only 49.39% of the global population is fully vaccinated [31], which is still some way short of the worldwide herd immunity target.

This study also tested the relationship between the COVID-19 vaccination rate with the proportion of new confirmed cases, dead cases, cases in hospital, ICU cases, and the reproduction rate of virus before and after the emergence of Omicron. From the perspective of disease defense, although large-scale vaccination is being globally implemented, new challenges occur and request the development of vaccines against mutant strains. In addition, vaccine inequity will bring more significant crises since regions without herd immunity have potential risks of virus mutation. Countries with lower vaccination coverage always have lower HDI, which places a heavy burden on the public health system of these countries. Research also argued that the virus is more likely to thrive and mutate in regions with low vaccination coverage [34], becoming a new problem and challenge for the global response to the epidemic.

### 4.3. How to Reduce Vaccine Inequity

Vaccine inequity has a long history, and studies found that vaccine inequity, as in the cases of influenza vaccine [35] and HPV vaccine [36], persisted in countries with different socioeconomic development levels. However, the COVID-19 is different from other epidemics in that it has impacted the global system and disrupted people’s daily life. As an essential measure to control the pandemic, the COVID-19 vaccine is a vital approach to restart the social system and counteract the epidemic. COVID-19 vaccine inequity will delay the fading of the virus [37], prolong the global crisis [9], undermine the recovery of the economy worldwide [38], and widen the development gap between countries [39]. Hence, it is urgent to address COVID-19 vaccine inequity.

The WHO has been implementing COVAX to solve the COVID-19 vaccine inequity, but it still needs a worldwide effort. For LMIC, they need to increase the incentive for vaccine access under the COVAX framework. For example, countries purchasing large vaccines have to exchange timely delivery schedules with COVAX and the African Vaccine Acquisition Trust (AVAT) to avoid vaccine stockpiling [40]. Furthermore, HIC needs to participate in the COVAX program actively and donate vaccines, having these as their promises. HIC has pledged to donate more than 1 billion doses of vaccine, but less than 15% has been delivered so far [41]. Vaccine producers can share vaccine development technologies and support local vaccine production in LMIC. Moreover, other organizations and enterprises can provide funds and vaccines to alleviate the financial difficulties of vaccination in LMIC. In addition, all countries need to remove export restrictions and any other trade barriers to the COVID-19 vaccine and the inputs involved in its production [42].

Tedros Adhanom Ghebreyesus stated that raising global awareness of fast-spreading respiratory pathogens is also essential for addressing vaccine inequity [42]. In addition to the financial and technological support, constructing the importance of vaccine equity at a conceptual dimension is necessary for response to the epidemic. Studies have confirmed that media plays a role in guiding the public to form the correct health beliefs and perceptions [43]. First, mass media need to report on the characteristics of the epidemics actively and illustrate the principles of spread, hazards, and response measures. Then, the media need to raise public awareness of the current vaccination situation worldwide and explain the risks of vaccine inequity in controlling the pandemic. Finally, public health departments can enhance the dissemination of information on vaccination and the epidemic dynamics via mass media and social media channels, providing official and authoritative sources of health information. The media needs to shoulder its role in raising public perceptions of the COVID-19 situation, vaccination, and vaccine inequity, promoting more effective public health communication.

## 5. Conclusions

Differences in HDI among countries caused COVID-19 vaccine inequalities worldwide. The study found that vaccination significantly mitigates the pandemic and reaching 70% immunization coverage can further control the epidemic. Thus, promoting COVID-19 vaccination further is necessary, especially in countries with low vaccination coverage. Moreover, improving the 70% immunization coverage worldwide shares the same significance with bridging vaccination gaps in controlling the pandemic. In addition, the occurrence of Omicron makes the COVID-19 epidemic situation even worse, suggesting the importance of further vaccine development and promotion. The globe will face a more significant challenge in controlling the pandemic without more vaccination coverage in lower-vaccinated countries. Addressing the issue of vaccine inequity needs the cooperation of HIC, LMIC, public health departments, and vaccine producers. Moreover, the media has to contribute to effective public health communication by raising public perceptions of the COVID-19 pandemic, vaccination, and vaccine inequity.

## 6. Limitations

This study examined COVID-19 vaccination and vaccine inequity from the perspective of data research, but the formation mechanism of vaccine inequity needs further exploration. In addition, the COVID-19 epidemic is still evolving, and global vaccination is in progress, so the relevant data and further changes in vaccine inequity need to be updated. In addition, the original data set did not distinguish between the types of COVID-19 vaccines received in different countries; thus, this empirical study did not take the type of vaccines into account.

## Figures and Tables

**Table 1:** Variable definition.

Variables Type	Variables	Definition
Vaccination	Total vaccinations per hundred	Total number of vaccines per hundred population
	People vaccinated per hundred	Number of people vaccinated per hundred population
Country difference	HDI	Human development index
COVID-19 epidemic situation/ Immunization Coverage	New cases per million	Number of new cases per million population
	New deaths per million	Number of new deaths per million population
	Hosp patients per million	Number of hospital patients per million population
	ICU patients per million	Number of ICU patients per million population
	Reproduction rate	Reproduction rate of the virus
Vaccinated rate	People vaccinated per hundred	Number of people vaccinated per hundred population
	Seventy	Seventy = 1 if People vaccinated per hundred ≥70; Seventy = 0 if People vaccinated per hundred <70

**Table 2:** Results of the testing model 2 with OLS.

	(1)	(2)
	Total Vaccinations per Hundred	People Vaccinated per Hundred
HDI	359.0256 ***	145.4545 ***
	(19.5340)	(17.9730)
Cons	−161.1684 ***	−56.9376 ***
	(−11.8609)	(−9.5162)
*N*	184	184
*R^2^*	0.6771	0.6396
Adj. *R^2^*	0.6753	0.6376

Note: z statistics in parentheses, *** *p* < 0.001.

**Table 3:** Results of the testing model 2 with fixed effects.

	(1)	(2)	(3)	(4)	(5)
	New Cases per Million	New Deaths per Million	Hosp Patients per Million	ICU Patients per Million	Reproduction Rate
People vaccinated per hundred	0.9714 ***	−0.0276 ***	−1.8835 ***	−0.3274 ***	0.0007 ***
	(3.6280)	(−7.3020)	(−10.2160)	(−13.6723)	(3.5287)
Cons	137.6359 ***	3.0265 ***	259.3673 ***	40.8461 ***	0.9693 ***
	(14.5898)	(23.0059)	(28.8352)	(35.2436)	(149.5766)
*N*	6543	6230	1239	1197	5771
*R* ^2^	0.0021	0.0088	0.0799	0.1386	0.0022
adj. *R*^2^	−0.0309	−0.0243	0.0523	0.1134	−0.0305

Note: z statistics in parentheses, *** *p* < 0.001.

**Table 4:** Results of the testing model 3 with fixed effects.

	(1)	(2)	(3)	(4)	(5)
	New Cases per Million	New Deaths per Million	Hosp Patients per Million	ICU Patients per Million	Reproduction Rate
Seventy	154.1341 ***	−0.7331 ***	−26.9153 *	−6.3113 ***	0.0570 ***
	(14.9727)	(−3.4238)	(−2.2412)	(−4.1022)	(3.8525)
Cons	91.2220 ***	1.6633 ***	155.9631 ***	22.8505 ***	1.0006 ***
	(40.9982)	(36.0432)	(39.3127)	(43.7744)	(328.4747)
*N*	13987	12319	2198	2079	11424
*R* ^2^	0.0160	0.0010	0.0023	0.0082	0.0013
adj. *R*^2^	0.0007	−0.0157	−0.0143	−0.0083	−0.0150

Note: z statistics in parentheses, * *p* < 0.05, *** *p* < 0.001.

**Table 5:** Results of the testing model 2 with fixed effects (test in separate groups).

	(1)	(2)	(3)	(4)	(5)
	New Cases per Million	New Deaths per Million	Hosp Patients per Million	ICU Patients per Million	Reproduction Rate
Panel A (9 November 2021)					
People vaccinated per hundred	−0.4758 *	−0.0421 ***	−2.6645 ***	−0.4037 ***	−0.0004 *
	(−2.3848)	(−8.4185)	(−13.6655)	(−15.6530)	(−2.0869)
Panel B (after 9 November 2021)					
People vaccinated per hundred	22.3892 **	−0.0556	−18.5514 **	0.7395	0.0281 ***
	(2.6758)	(−0.9959)	(−3.0328)	(1.3872)	(5.1115)

Note: z statistics in parentheses, * *p* < 0.05, ** *p* < 0.01, *** *p* < 0.001.

## Data Availability

Publicly available datasets were analyzed in this study. The data can be found here: https://ourworldindata.org/coronavirus (accessed on 20 April 2022).
